# Evaluating the taxa that provide shared pollination services across multiple crops and regions

**DOI:** 10.1038/s41598-019-49535-w

**Published:** 2019-09-19

**Authors:** Bryony K. Willcox, Brad G. Howlett, Andrew J. Robson, Brian Cutting, Lisa Evans, Linley Jesson, Lindsey Kirkland, Malou Jean-Meyzonnier, Victoria Potdevin, Manu E. Saunders, Romina Rader

**Affiliations:** 1 School of Environmental and Rural Science, University of New England, Armidale, NSW Australia; 2 The New Zealand Institute for Plant and Food Research Limited, Private Bag 4704, Christchurch Mail Centre, Christchurch, 8140 New Zealand; 3 Precision Agriculture Research Group, School of Science and Technology, University of New England, Armidale, NSW Australia; 4 Plant & Food Research Australia, Queensland University of Technology, M Block Room 581, Gardens Point Campus GPO Box 2434, Brisbane, 4001 Australia; 5 The New Zealand Institute for Plant & Food Research Limited, Hawke’s Bay, Crosses Rd, Parkvale, Havelock North 4172 New Zealand; 6 Agrocampus Ouest, Rennes, France; 7 UNE Business School, University of New England, Armidale, NSW Australia

**Keywords:** Agroecology, Ecosystem services

## Abstract

Many pollinator species visit multiple crops in multiple regions, yet we know little about their pollination service provisioning at local and regional scales. We investigated the floral visitors (n = 13,200), their effectiveness (n = 1718 single visits) and response to landscape composition across three crops avocado, mango and macadamia within a single growing region (1 year), a single crop (3 years) and across different growing regions in multiple years. In total, eight wild visitor groups were shared across all three crops. The network was dominated by three pollinators, two bees (*Apis mellifera* and *Tetragonula* spp.) and a fly, *Stomorhina discolor*. The visitation network for the three crops was relatively generalised but with the addition of pollen deposition data, specialisation increased. Sixteen managed and wild taxa were consistently present across three years in avocado, yet their contribution to annual network structure varied. Node specialisation (*d*’) analyses indicated many individual orchard sites across each of the networks were significantly more specialised compared to that predicted by null models, suggesting the presence of site-specific factors driving these patterns. Identifying the taxa shared across multiple crops, regions and years will facilitate the development of specific pollinator management strategies to optimize crop pollination services in horticultural systems.

## Introduction

Wild and managed pollinator taxa (including bees, flies, beetles, butterflies and moths) provide and stabilise pollination services throughout agricultural and natural systems^1–3^. Yet, high inter-annual variation in the abundance and efficiency of wild taxa^4,5^ and pest and disease pressures on populations of honey bees (*Apis mellifera* L.)^6^ are ongoing challenges to consistent pollination service delivery. Identifying the taxa shared across multiple crops, regions and years is important to support key taxa that optimise crop pollination services at broader scales in agricultural systems^7,8^. Hence, an improved understanding of how to effectively manage both wild and managed taxa together will likely inform management practices that provide more efficient and resilient crop pollination services to growers^1,9^.

While there has been a focus on managing and increasing abundances of wild bee taxa in crop systems, pollinator communities with both bee and non-bee taxa benefit crops in several ways. Taxonomically rich pollinator communities are more resilient to fluctuating population dynamics and species extinctions due to their capacity to provide functional redundancy and complementarity^10–13^. In agricultural crops, functional redundancy is the result of numerous potential pollinators at a local or regional scale^12,14^. Diversity in pollinator and plant communities, enables redundancy and complementarity in pollinator assemblages which can improve their functioning^15,16^. For example, flies often have a wider temporal range of activity compared to bees^17,18^ and the abundance of some fly species is influenced by agricultural intensification^19^. Therefore, diverse communities of bee and non-bee pollinators that together provide pollination services irrespective of the time of day will ensure crops with highly variable receptivity periods will be pollinated^20^. Diverse assemblages and a high turn-over of pollinator species between seasons or across years and spatial scales are thus important to support yields in pollinator-dependent crop systems^10–12^.

Existing management strategies often focus on supporting wild bee populations through the provision of floral and nesting resources. These include the maintenance of semi-natural vegetation within agricultural landscapes, planting annual floral strips^21,22^ and targeted perennial plantings^23,24^. In addition to these, mass-flowering crops can also provide temporary foraging resources within some mosaic landscapes^25–28^ but the benefits seemingly depend on crop flowering phenology, landscape composition and configuration^28–32^. For example, some studies have found landscape heterogeneity to have a positive effect on wild bee species richness and seed set of sentinel plants, but other studies have found little evidence^31–35^. Furthermore, the phenology of mass flowering crops can influence regional bee diversity and crop yields in other sequentially flowering crops^28–30^. Greater research effort is required to understand the impact of mass flowering crops on non-bee pollinators as well as the influence of landscape composition on pollinator-crop and pollinator-site network structures.

Although a mix of wild and managed pollinating taxa is a prudent strategy, we still know relatively little about (i) the identity of wild pollinators that visit multiple co-flowering or sequentially flowering crops across a number of growing regions and (ii) the level of specialisation of pollinator taxa on crops across different habitats. Quantification of the connections between plants and pollinators^36–38^, species and habitats^39^, and crops and pollinators^28^ is urgently required in order to devise strategies that support the *in-situ* management of local, wild pollinator taxa across multiple crops and regions. This would provide a focus for management efforts on local, wild pollinator taxa that already exist in a given landscape, to support or augment managed honey bee services, without importing new exotic taxa and their associated pests, parasites and disease^1–3,12,40–42^.

We surveyed insect visitors and evaluated their effectiveness by constructing crop-pollinator networks in three tree crops grown in Australia to identify common taxa and to understand how co-flowering crops, annual variation and landscape factors influence crop-pollinator network structure. Specifically, we ask the following questions:

1. Which insect pollinator taxa are shared between co-flowering crops within a single growing region in one year?
2. Are insect pollinator taxa shared across different growing regions in a given crop?
3. How do crop-pollinator networks in a single crop vary across years?
4. How does landscape composition influence site level structure within crop-pollinator networks?

## Results

### Shared pollinator taxa across crops

A total of 13,200 flower visiting insects were observed in Bundaberg (6,311), Mareeba (3,149) and Sunraysia (3,740). Honey bees (*Apis mellifera*) comprised 26% of total visits and were found in all crops, regions and years. Managed honey bee hives were present at or within flight range of all orchards and feral honey bee colonies were also observed close to several orchard blocks. Two families of fly visitors, Syrphidae and Calliphoridae, were present across all regions and crops. A single genus belonging to Calliphoridae (*Chrysomya* spp.) was present in all crops and regions, comprising 5% of total visits (See SI Tables 1, 2 for further information regarding other genera present in crops across regions).

**Figure 1:**
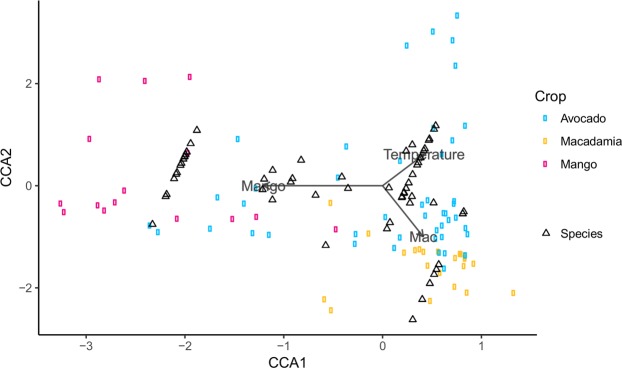
Canonical correspondence analysis plot showing the distribution of pollinator taxa and orchard sites as a function of crop type (avocado, macadamia and mango) in Bundaberg, 2016. Grey arrows show significant explanatory factors with length of arrow indicative of strength of significance.

In the Bundaberg region alone, 2,882 flower visitors were observed across avocado (1,663), macadamia (719) and mango orchards (500) in 2016. The constrained variables in the canonical correspondence analysis (CCA) explained 13% of the variation in pollinator community composition. The variance explained by crop type (11%, *p* = *0*.*001*) and temperature (2%, *p* = *0*.*021*) was significant (Fig. 1), while the variance explained by CCA1 (7%, *p* = *0*.*001*) and CCA2 (4%, *p* = *0*.*002*) was also significant.

**Figure 2:**
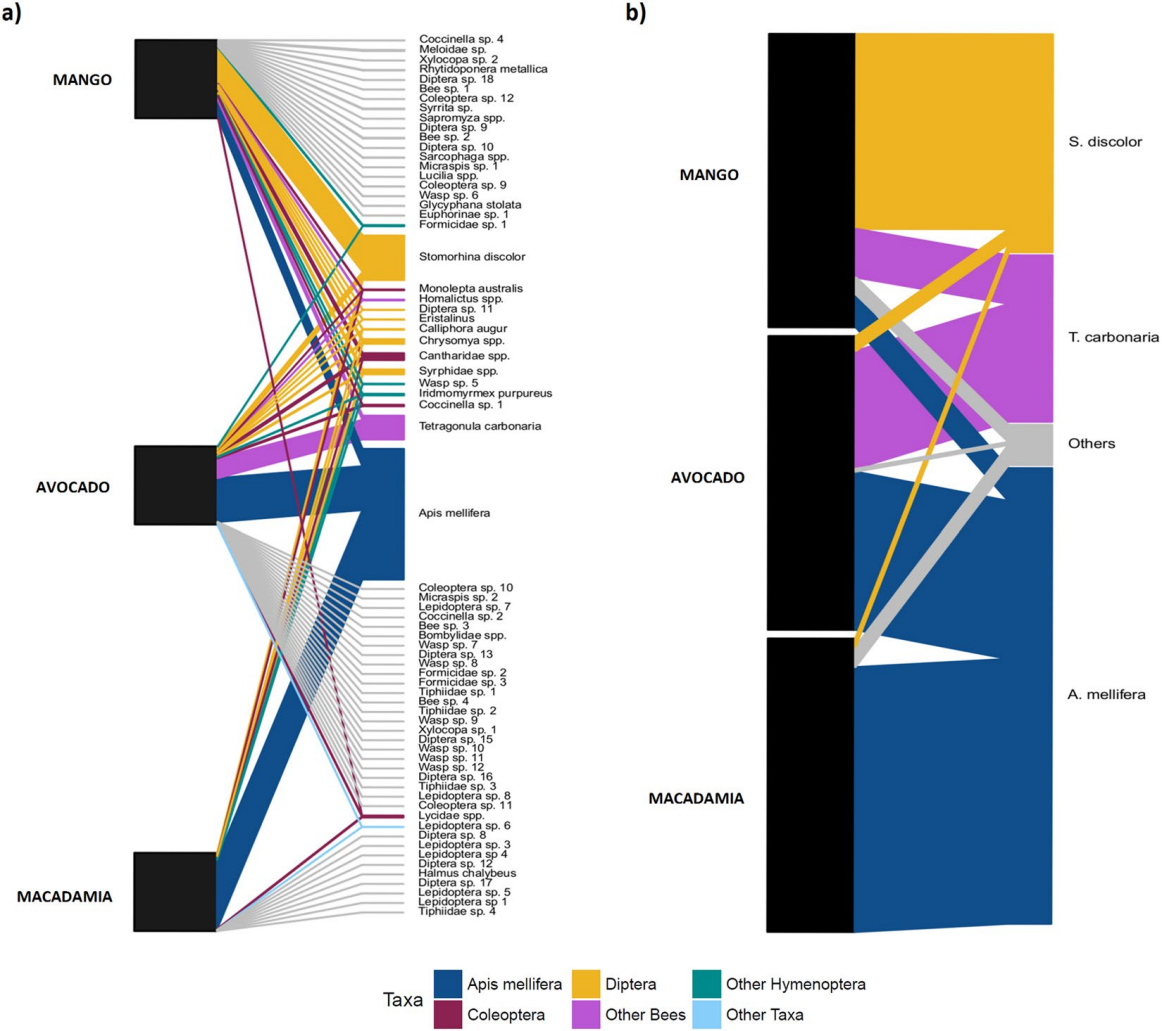
Insect visitor networks of mango, avocado and macadamia orchard sites in Bundaberg during flowering in 2016; (**a**) visitation network and (**b**) pollinator effectiveness network. Pollinator species shared between crops shown in colour and coded to different taxa. Height of each visitor group node reflects proportional abundance.

In total, eight wild visitor groups (including *Chrysomya* spp.) were shared across all three crops (Fig. 2a). With a further seven groups shared between avocado and mango crops and a single group (Lepidoptera sp. 1) was shared between avocado and macadamia. The network was dominated by three pollinator groups including *Apis mellifera* (species strength = 1.67), *Stomorhina discolor* (species strength = 0.57) and *Tetragonula* spp. (species strength = 0.31), although *Tetragonula* spp. were not observed in macadamia orchards.

The visitation network for the three crops was relatively generalised (*H2*′ = 0.41, Fig. 2a), but with the addition of pollen deposition data, in the pollinator effectiveness network, specialisation increased (*H2*′ = 0.59, Fig. 2b). The inclusion of pollen deposition data did not alter the ranking of pollinator species strength from that observed in visitation networks for avocado and macadamia crops. However, in mango, native stingless bees *Tetragonula* spp. contributed more to pollination services, despite visiting less frequently than honey bees. Pairwise comparisons of pollinator species strength between visitation and pollination effectiveness networks revealed no significant differences.

### Shared insect pollinator taxa across different growing regions

**Figure 3:**
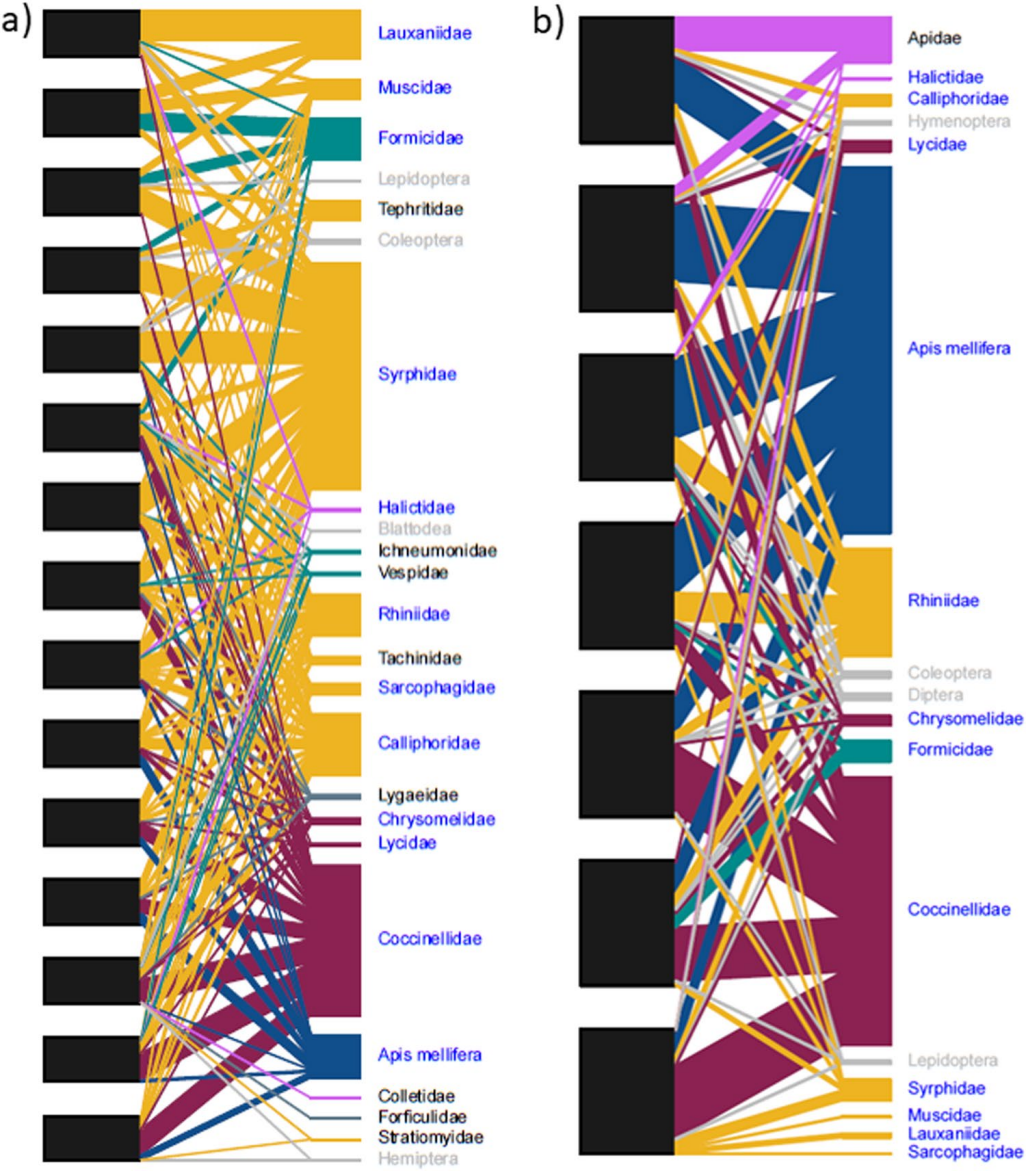
Family level visitation networks of avocado orchard sites in (**a**) Sunraysia region [15 orchard sites], and (**b**) Bundaberg [7 orchard sites] during flowering in 2015. Black nodes indicate orchard sites in each region. Visitor group labels colour coded to indicate shared groups (blue), unique groups (black) and unidentified visitors grouped to order (grey).

Visitation networks revealed several shared families between growing regions for avocado (11 families; Fig. 3) and mango (8 families; SI Fig. 1). However, species strength metrics indicated large variations in important pollinator taxa between regions. Networks for both crops in Bundaberg (*H2*′ values: avocado = 0.21 and mango = 0.13) were more generalised when compared to Sunraysia (avocado *H2*′ = 0.36) and Mareeba (mango *H2*′ = 0.39). The most important taxa in each region varied for both avocado (Bundaberg *A*. *mellifera* [species strength = 2.93] and Sunraysia Syrphidae [species strength = 5.23]) and mango (Bundaberg Rhiniidae [species strength = 0.97] and Mareeba Apidae [*excludes Apis mellifera*, species strength = 1.72]).

### Variation in crop-pollinator networks in a single crop across years

Network interactions (*H2*′) across all three years in avocado (Bundaberg, QLD) were relatively generalised (2015 = 0.25, 2016 = 0.36 and 2017 = 0.20; SI Fig. 2) indicating these orchard sites are mostly visited by the same common species. Eight visitor taxa were present across all three years, with species strength metrics indicating variation in taxa importance across years. In 2015, a morphospecies of coccinellid beetle was significantly important in comparison to null model predictions (species strength = 1.36, *p* = 0.015), while in 2017, a species of fly, *Stomorhina discolor* was marginally important (species strength = 1.04, *p* = 0.046). *Apis mellifera* was significantly important in each of the three years (2015 - species strength = 2.93, *p*  < 0.001; 2016 - species strength = 4.24, *p*  < 0.001; 2017 species strength = 2.3, *p* < 0.001) (SI Fig. 3).

### Landscape composition and crop-pollinator networks

Specialisation (*d*’) values of orchard sites in all seven networks indicated they were typically relatively generalised in their pollinator network structure, with most values being below 0.5 (SI Figs 4–7). Despite this, null models predicted significantly more generalised structures for most sites within these systems. Avocado sites in Bundaberg were more specialised when there was a higher proportion of native forests and vegetation within a 2 km radius (native forests *t* = 3.6, *p* = 0.04; native vegetation *t* = 3.89, *p* = 0.03) (Table 1). Mango sites in Mareeba were more specialised with higher proportions of irrigated crops within a 1 km radius (*t* = 4.27, *p* = 0.008), and macadamia sites in Bundaberg were more generalised with higher proportions of irrigated cropping within a 1 km radius (*t* = −4.34, *p* = 0.049) (results for remaining perimeters in SI Table 3).

**Table 1:** Minimum adequate GLM models describing the influence of landscape composition on orchard site specialisation (*d*’).

Predictor variables	Estimate	Std. Error	t value	Pr(>|t|)
**Avocado – Bundaberg**, **2015**, **2 km**				
(Intercept)	−3.678	0.626	−5.87	*0*.01*
Irrigated cropping	0.023	0.009	2.6	0.08
Native forests (production)	0.034	0.01	3.6	*0*.04*
Native vegetation (grazing)	0.027	0.007	3.89	*0*.03*
**Macadamia – Bundaberg**, **2016**, **1 km**				
(Intercept)	−0.839	0.655	−1.28	0.329
Native vegetation (grazing)	−0.007	0.008	−0.85	0.48
Irrigated perennial horticulture	−0.02	0.007	−2.844	0.105
Irrigated cropping	−0.029	0.007	−4.344	*0*.*049*
**Mango – Mareeba**, **2016**, **1 km**				
(Intercept)	−2.185	0.743	−2.941	*0*.032*
Native vegetation (grazing)	0.023	0.01	2.36	0.065
Irrigated perennial horticulture	−0.008	0.01	−0.548	0.608
Irrigated cropping	0.086	0.02	4.265	*0*.008**

## Discussion

Using multi-crop, multi-year and multi-region crop-pollinator networks we demonstrate here that shared wild and managed pollinator taxa visit multiple crops across several regions. In particular, we found honey bees (*A*. *mellifera*) and two families of wild visitors, Syrphidae and Calliphoridae, are common across all regions and crops. Further, regional comparisons for both avocado and mango crops identified additional shared families that were locally abundant such as beetles (Coccinellidae) and native Apidae (*Tetragonula* spp.). These results suggest that there is significant potential to identify shared pollinators that provide services across multiple crops and use this knowledge to develop pollination management strategies that focus on the resource needs of these wild taxa.

Species strength values indicated the visitation network across avocado, macadamia and mango crops in the Bundaberg region was dominated by three shared species, including managed honey bees (*A*. *mellifera*), a fly species, *S*. *discolor* and the native bee, *Tetragonula* spp. When pollen deposition measures were incorporated for each of these species, these three accounted for the majority of the crop pollination service provisioning. Our results support previous studies that indicate dominant pollinator taxa provide the majority of the pollination service provisioning in many crops^43^ and visitation networks are a good proxy for the pollination effectiveness of these more frequently interacting species^44^. Pollinator effectiveness rankings only varied in mango whereby the effectiveness of *Tetragonula* spp., a native stingless bee, was greater than the more frequent visitor, *A*. *mellifera*. While the use of conservative pollen deposition estimates for rare and unique species observed potentially underestimates their effectiveness in individual crops; it nonetheless highlights their role in providing pollination services to these crops as well as the need for additional research into more fully understanding their overall contribution.

Inter-annual variation in the structure and species importance observed in avocado-pollinator networks in one region highlights the importance of diverse communities in facilitating crop pollination services over successive seasons. While the dominant pollinator species, managed *A*. *mellifera*, was consistent across all three years, several co-occurring common wild species including *Coccinella* sp., *S*. *discolor*, and *Tetragonula* spp. were also present in each year of the study. Two of these species, *Coccinella* sp. (in 2015) and *S*. *discolor* (in 2017), were significantly important within the networks. While similar temporal consistency in common, unmanaged pollinator taxa has been found in other crops, e.g. *Brassica rapa* in New Zealand^45^, temporal studies across years that investigate pollinator network variations of co-flowering crops are lacking. We advocate that a greater understanding of variation in pollinator networks and the impact on pollination effectiveness and yield across multiple crops and successive seasons is an important future research priority.

Node specialisation (*d*’) analyses indicated many individual orchard sites across each of the networks were significantly more specialised than predicted by null models, suggesting the presence of site-specific influencing factors driving these patterns. Few clear trends emerged when surrounding land use type was used to explain variation in site level specialisation. This may be due to a mismatch between a more complex pollinator network and a relatively simple, single-scale definition of the surrounding landscape heterogeneity, rather than the absence of any influence. Previous studies that used measures of species richness and abundance to investigate local variations among crop sites found groups of taxa, such as wild bee pollinator richness and abundance, were strongly positively correlated with the proportion of semi-natural habitat surrounding crop sites^46,47^. More variable responses have been observed with increasing proportions of cropping habitat^48^. For pollinator networks that include many different bee and non-bee taxa, all of which may respond in different ways to various environmental conditions and potentially to factors at multiple landscape scales^27^, the relationship between network structure and these factors may be considerably more complex. For example, patterns of landscape influence on non-bee pollinator taxa are far more varied^15,49,50^ and their response to changes in semi-natural habitat is less pronounced compared to wild bees^3^. In addition, honey bees (*A*. *mellifera*) show little or no response to the proximity of semi-natural or natural habitat^3,51^, but may forage outside the target crop species dependent upon their pollen or nectar preferences and the needs of the hive^52,53^. Given the abundance of non-bee taxa across our networks, a greater understanding of the habitat and floral resource requirements these taxa have outside of crop flowering windows is needed. The development of pollinator management plans that incorporate food and habitat preferences of diverse pollinator communities will also facilitate our understanding of suitable landscape configurations.

While common species were identified, pollinator assemblage composition varied significantly between co-flowering crops, largely due to varying proportions of shared groups and the presence of many rare species unique to each crop. Variation in node (species) strength, and in network structure over time, suggests there is much variation in the contribution to pollination among regions. These findings support previous studies that confirmed pollinator diversity and variation in space and time are clearly important to crop production^12,13^. Thus, we caution a focus solely on a few common species. Rather our results suggest that the identification of selected taxa could enable prioritization of management strategies to move toward the management of wild taxa in agricultural landscapes. Management of common shared species in combination with other taxa is an important strategy to reduce risk and maximize resources to ensure ongoing and efficient crop production across space and time^9^. In particular, the abundance of non-bee taxa across our networks emphasises the need to further understand the life-cycle requirements of these taxa, including how varying conditions from year to year affect them^17^. Further, geographic and other boundaries will limit the occurrence of particular families or species being shared between regions, hence, prioritizing the management of local taxa in a given region may be more relevant to particular crops with respect to the development of pollinator management plans.

Developing effective management strategies that account for both managed and wild insect pollinators to ensure ongoing crop pollination services is a current global priority. The findings from this study highlight the need for strategies that are region-specific and inclusive of both non-bee taxa and co-flowering crop vegetation. Furthermore, the inclusion of non-bee pollinator taxa into management strategies will require additional research to identify factors that influence pollinator communities at an orchard site-level.

## Methods

### Study sites

Study sites were selected within commercial avocado (*Persea americana*), mango (*Mangifera indica*) and macadamia (*Macadamia integrifolia*) orchards in three major growing regions of Australia (SI Fig. 8). These crops are grown predominantly in subtropical and tropical areas of Queensland, New South Wales, Western Australia and the Northern Territory with avocado also produced in South Australia, Victoria and Tasmania. Avocado sites (cv. Hass) were selected in the Sunraysia region [including; Renmark, South Australia (34.1743°S, 140.7443°E); Mildura, Victoria (34.2080°S, 142.1246°E), and Coomealla, New South Wales (34.1129°S, 142.068°E)] and Bundaberg, Queensland (24.8670°S, 152.3510°E). Mango sites (cv. Keitt) were surveyed in Mareeba, Queensland (17.0019°S, 145.4389°E) and (cv. Calypso) in Bundaberg, Queensland (24.8670°S, 152.3510°E). Macadamia (cv. 741) was surveyed in Bundaberg only. The number of sites varied across regions, between crops and years (SI Table 4). In Bundaberg, all three crops overlapped in flowering phenology with mango generally the first to flower starting in early to mid-August (late winter) followed by avocado and macadamia in late August - early September (late winter - early spring). To ensure adequate pollination throughout the flowering period all growers placed managed honey bee hives within or near to orchard blocks^54,55^.

### Pollinator surveys

Pollinator visitation surveys were conducted at commercial orchard study sites within each region. The minimum distance between sites within a region was 1 km, with the exception of the two mango sites in Bundaberg. Survey methods involved observing insect visitation to flowers on 10–18 trees either in a straight transect or spread throughout an orchard block; further details about survey methods and recording weather variables are provided in Howlett *et al*.^56^. Observation surveys were conducted between 3 and 6 times per day and totalled 20 minutes per observation. All flower visiting insects were recorded during these observations. Insect visitors were identified to visitor group (based on flight behaviour and morphology) in the field and a reference collection for each region was used to maintain consistency between sites and observers. Following the identification of voucher specimens, some visitor groups contained multiple genera or species. Analyses were conducted on visitor groups observed in the field, but information regarding genera and species that comprised these groups is included in the supplementary information (SI Table 5). These specimens were then deposited with the Rader Pollination Laboratory, University of New England, Armidale, Australia. Surveys were carried out in all weather conditions, excluding rain (SI Table 4).

### Crop pollination effectiveness

Pollen deposition by insects onto floral stigmas was estimated via single visit deposition (SVD) experiments. SVD was measured using an active approach, which entailed detaching virgin flowers (bagged to prevent insect visitations) and offering these to a target pollinator species foraging in each crop^57^. Despite the potential for pollinators to behave differently or even be put-off by the use of manipulated flowers, most species we targeted accepted the manipulated flower. These experiments coincided with pollinator surveys and were conducted at the same orchard sites. Target insects were identified in the field using the visitor group categories defined for pollinator surveys. Following a single visit, stigmas were immediately removed from flowers and mounted onto slides. To make up slides, the stigmas were excised and placed on a cube of gelatine-fuchsin, a coverslip was placed on top of the gelatine cube and gentle heat was applied to melt the gelatine^58^. Stigmatic pollen loads were then estimated by counting either avocado, mango or macadamia pollen grains under 200x magnification. We also collected a second virgin control stigma (bagged and unvisited) allowing us to assess other potential sources of pollen to stigmatic totals^5^. Across the three crops, SVD data was collected over 4 years between 2014 and 2017.

For pollen data analysis, we opted to use the most conservative estimate available with our first choice being the median of pollen deposition estimates^59^. Due to the distribution of the pollen deposition values, the median value in our data sets was most often zero, hence we opted to use mean deposition values. We accounted for non-insect related pollen deposition on each stigma, with the mean pollen value from the collected control stigmas for each data set subtracted from individual SVD values for each insect taxa^60^ (SI Table 6). We calculated ‘pollinator effectiveness’ by multiplying visitation rate and mean SVD for each field defined visitor group to gain an understanding of their pollination effectiveness in a given crop^9,60,61^. Pollen deposition information was not collected for rare or less abundant visitor species observed in our visitation networks. However, rather than disregarding their contribution we grouped these visitors together and multiplied their visitation rate by the lowest mean deposition value from an insect taxon collected to represent the most conservative estimate of their effectiveness possible.

### Landscape composition

Landscape composition was defined as the proportion of different land uses around each site and was quantified with ArcMap 10.2 based on state and national land use mapping data sets^62–64^ using the Australian Land Use and Management (ALUM) 3-tier land use classification system (SI Fig. 9). To standardise our use of land type definitions in this study we selected second tier ALUM classifications that could potentially provide resources (floral and nesting) for pollinator taxa surrounding our study orchard sites. Land use types were quantified for three perimeters (500 m, 1 km and 2 km) surrounding orchard sites where overlap in perimeters did not occur (i.e. several mango sites in Bundaberg and avocado sites in Sunraysia were excluded from these analyses due to their close proximity) in all three regions and years corresponding to observation surveys. This perimeter gradient was chosen due to the varying spatial scales at which insect taxa are influenced by landscape factors, for example wild bee species are known to be influenced by smaller spatial scales up to 1 km^65^, while fly species are influenced by varying spatial scales^15^. In Bundaberg and Mareeba, the dominant land types quantified were native vegetation (used for grazing animals), irrigated cropping (predominantly sugar), irrigated perennial horticulture (tree fruits, tree nuts and vegetables) and native forests (used for production purposes), with no natural conservation areas within these perimeters. In the Sunraysia region, the landscape was less heterogenous than Bundaberg and Mareeba, with only three land use types available to quantify including native vegetation (used for grazing animals), irrigated perennial horticulture (tree fruits, tree nuts and vegetables) and natural conservation areas (e.g. National parks, other protected natural areas).

### Data analyses

To assess shared pollinator taxa between multiple crops within a single growing region and year, we focused on three co-flowering crops in the Bundaberg region: macadamia, avocado and mango. A canonical correspondence analysis (CCA), with crop type and temperature as explanatory variables, was initially undertaken so that differences in pollinator communities between crops could be visualised.

**Figure 4:**
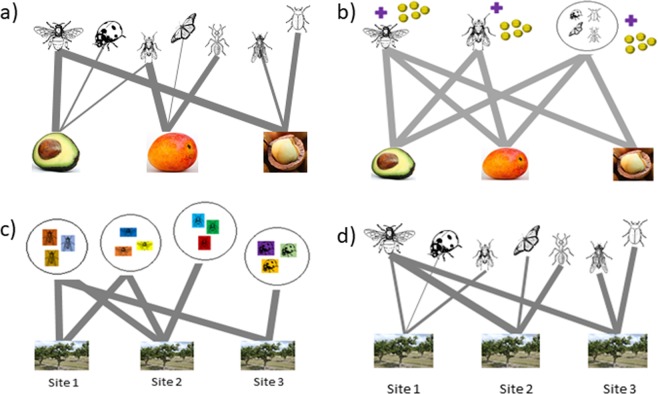
Visual representations of bipartite networks, highlighting different node types used in this study. Question 1 networks; (**a**) visitation network with crop type and insect visitor group as nodes; (**b**) pollinator effectiveness network with crop type and insect visitor group (visitation rate x mean pollen deposition) as nodes. Questions 2 and 4 networks; (**c**) regional visitation networks with individual orchard sites and taxonomic families as nodes; (**d**) visitation networks with individual orchard sites (unique crop, region and year) and insect visitor group as nodes. Question 3 networks.

We then constructed two bipartite networks with crop type linked to pollinator taxa as nodes: one network (i.e. pollinator visitation network; Fig. 4a) was weighted with proportional visitation rates (visits per minute) and the other network (i.e. the pollinator effectiveness network; Fig. 4b) was weighted with proportional pollinator effectiveness (which we define as visitation per minute multiplied by mean SVD values for individual taxa) values. We focused on metrics that were known to be robust against variations in sampling effort, network size, total number of interactions and high proportions of singleton observations^66,67^. Using the ‘networklevel’ function in the bipartite package^68^, we calculated *H2*′, which describes the degree of partitioning among sites within an entire network and provides a method for comparing the degree of specialisation across different networks^66,67^. Larger *H2*′ values (relative to other networks) indicate greater selectivity among the species in the network. Using the ‘specieslevel’ function in the bipartite package^69^, we calculated node specialisation (*d*’) for lower nodes and species strength for upper nodes (also called node strength) in each network. Node specialisation (*d*’) values range between 0 (highly generalised) to 1 (highly specialised)^66^. For our analysis, a crop with low *d*’ would mostly interact with pollinator taxa that are common across the region, while crops with high *d*’ would mostly interact with taxa not found in other crop types. Species strength identifies key influential nodes in the network, where higher values indicate pollinator taxa that are important for crops in the region. To identify if pollinator taxa differed in their network roles between visitation and pollination effectiveness networks, a pairwise comparison of species strength scores was conducted utilising the ‘emmeans’ package in R^70^.

To evaluate which taxonomic families visited the same crop grown in different regions, we focused on two crops that were surveyed in two different regions in the same year: avocado (cv. Hass) in Bundaberg and Sunraysia (2015); and mango in Bundaberg (cv. Calypso) and Mareeba (cv. Keitt) (2016) (n = 4 networks). For each crop in each region, we constructed bipartite networks linking orchard sites with pollinator family taxa (Fig. 4c). We calculated *H2*′ and mean taxa node specialisation (*d*’) for each network.

To assess inter-annual variations in crop-pollinator networks in a single crop, we focused on avocado orchards in Bundaberg, the only crop-region combination to be sampled across three consecutive years. We constructed bipartite networks linking orchard sites in avocado to insect visitor taxa in 2015 (n = 7 sites), 2016 (n = 7 sites) and 2017 (n = 5 sites) (Fig. 4d). Networks were weighted by proportional visitation rates. We calculated *H2*′ for each network, to identify how interactions across networks varied between years. Species strength for each pollinator taxa was calculated to identify pollinator importance in different years. Null models were constructed for each of the networks; we then compared observed species strength measures to determine whether individual insect visitor taxa were more important than that expected given random interactions. Null models were built using the “nullmodel” and “vaznull” functions from the Bipartite package in R^68,71^. The “vaznull” function randomises the total number of individual interactions observed in the original matrix and produces null model networks where the main constraint is that connectance (number of links in the matrix) will be the same as in the observed network^71^. Z-scores [observed − mean (null)/standard deviation (null)] were then calculated and used to determine the significance of observed species strength metrics.

To assess how landscape composition surrounding orchard sites influenced crop-pollinator networks, we constructed separate bipartite networks with orchard sites and pollinator groups as nodes for each of the seven unique crop, region and year combinations used in this study. Null models were first used to understand how observed orchard site nodes within a pollinator network varied in their specialisation (*d*’) level from that expected given random interactions between species within crops and regions^72^. The construction of null models followed the same method as outlined for question 2, with the use of the ‘vaznull’ and ‘nullmodel’ functions from the Bipartite package in R^65,66^. Z-scores were then calculated and used to determine the significance of observed node specialisation (*d*’). To understand if observed orchard site *d*’ was influenced by surrounding land use types we used generalised linear models (GLMs) with a quasi-binomial error distribution to account for under-dispersion. For each of the three perimeters (500 m, 1 km, 2 km) at which land use type was quantified, separate models were built for each network. Each model was simplified to include only explanatory variables that contributed at least marginally (*p* ≤ 0.10) to model fit. Optimal models were selected using model comparisons conducted using the function *anova* in R. Spatial autocorrelation was assessed for both raw data and model residuals using the Moran I function in the R package “ape”, version 5.1^73^. No evidence of significant spatial autocorrelation was found between any of the sites, and thus sites were considered to be spatially independent in further analyses. All statistical analyses were conducted using R Statistical Software^74^.

## Supplementary information

Supplementary Information

## Data Availability

The datasets generated and analysed during the study are available from the Dryad Digital Repository 10.5061/dryad.hj627cr.
