# Supplementary material for: Evaluating the taxa that provide shared pollination services across multiple crops and regions
Source: Sci Rep. 2019 Sep 19;9:13538. doi: 10.1038/s41598-019-49535-w (PMC6753147; doi:10.1038/s41598-019-49535-w)
Supplement: Supplementary file 1 — Supplementary Information [file 41598_2019_49535_MOESM1_ESM.pdf]

Supplementary Information

Evaluating the taxa that provide shared pollination services across multiple crops and regions

Bryony K. Willcox[1*], Brad G. Howlett[2], Andrew J. Robson[3], Brian Cutting[4], Lisa Evans[4], Linley Jesson[5], Lindsey Kirkland[1], Malou Jean-Meyzonnier[6], Victoria Potdevin[6], Manu E. Saunders[1,7] and Romina Rader[1]

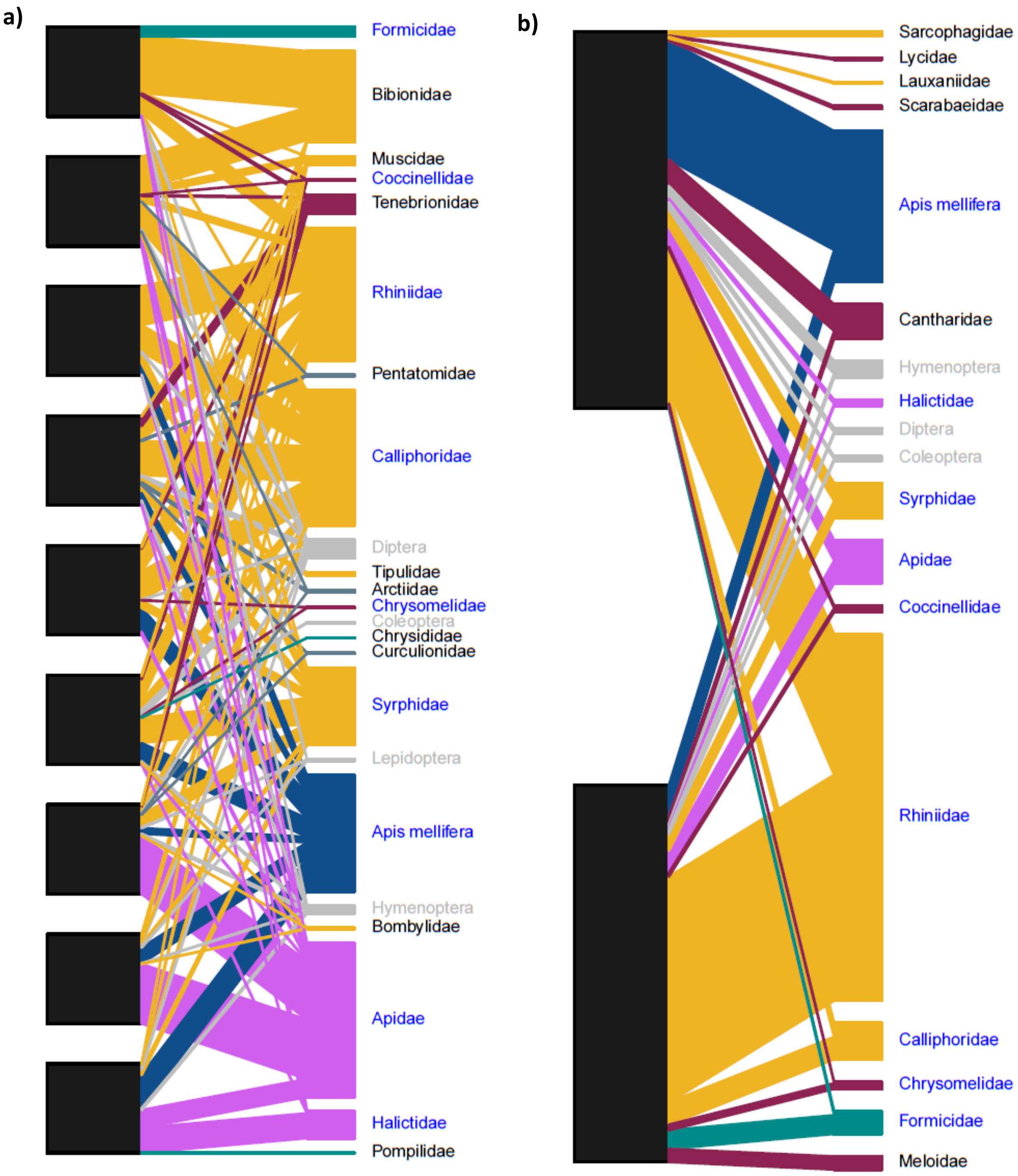

SI Figure 1: Family level flower visitor networks of mango orchard sites in a) Mareeba, QLD and b) Bundaberg during flowering in 2016. Black boxes indicate orchard sites. Visitor group labels colour coded to indicate shared groups (blue), unique groups (black) and unidentified visitors grouped to order (grey).

SI Table 1. Genera of shared families present in avocado across regions.

	Bundaberg (Queensland)		Sunraysia (New South Wales/Victoria/South Australia)	
**Taxonomic family**	**Species Strength**	**Genera present**	**Species Strength**	**Genera present**
Calliphoridae	0.7	*Chrysomya, Lucilia*	1.3	*Chrysomya, Lucilia, Calliphora*
Chrysomelidae	0.9	*Monolepta*	0.11	*Monolepta*
Coccinellidae	2.14	*Coccinella, Halmus, Micraspis*	3.31	*Coccinella, Harmonia*
Formicidae	0.4	*Iridomyrmex, Rhytidoponera* +	0.9	*Iridomyrmex, Rhytidoponera* +
Halictidae	0.14	*Lassioglossum*	0.03	*Lassioglossum*
Lauxaniidae	0.14	-	1.1	-
Lycidae	0.86	*Lycid*	0.03	*Lycid*
Muscidae	0.14	*Helina*	0.43	*Australphyra, Helina* +
Rhiniidae	0.85	*Stomorhina, Chlororhinia*	0.9	*Stomorhina, Metallea*
Sarcophagidae	0.14	*Sarcophaga*	0.22	*Sarcophaga*
Syrphidae	0.86	*Eristalinus, Simosyrphus Neoplesia, Cyphipeltus*	4.99	*Eristalinus,Simosyrphus,Neoplesia*
Managed Apidae	2.9	*Apis mellifera*	0.93	*Apis mellifera*

- specimens not identified any further than family

+ additional specimens present, not identified to genus

SI Table 2. Genera of shared families present in mango across regions.

	Bundaberg (Queensland)		Mareeba (Queensland)	
**Taxonomic family**	**Species Strength**	**Genera present**	**Species Strength**	**Genera present**
Apidae	0.11	*Tetragonula, Xylocopa*	1.72	*Tetragonula, Xylocopa, Apis**
Calliphoridae	0.1	*Chrysomya, Calliphora, Lucilia*	1.5	*Chrysomya, Lucilia, Metallea*
Chrysomelidae	0.02	*Monolepta*	0.01	*Monolepta*
Coccinellidae	0.02	*Coccinella, Micraspis*	0.01	*Coccinella*
Formicidae	0.06	*Iridomyrmex, Rhytidoponera* +	0.1	-
Halictidae	0.02	*Homalictus*	0.3	*Homalictus, Hylaeus*
Rhiniidae	0.97	*Stomorhina*	1.5	*Stomorhina*
Syrphidae	0.1	*Eristalinus, Simosyrphus Neoplesia, Cyphipeltus*	0.9	*Eristalinus, Allobacha, Allograpta Mesembrius, Melanstoma, Orthoprosopa*
Managed Apidae	0.4	*Apis mellifera*	1.3	*Apis mellifera*

- specimens not identified any further than family

+ additional specimens present, not identified to genus

* feral *Apis cerana* present

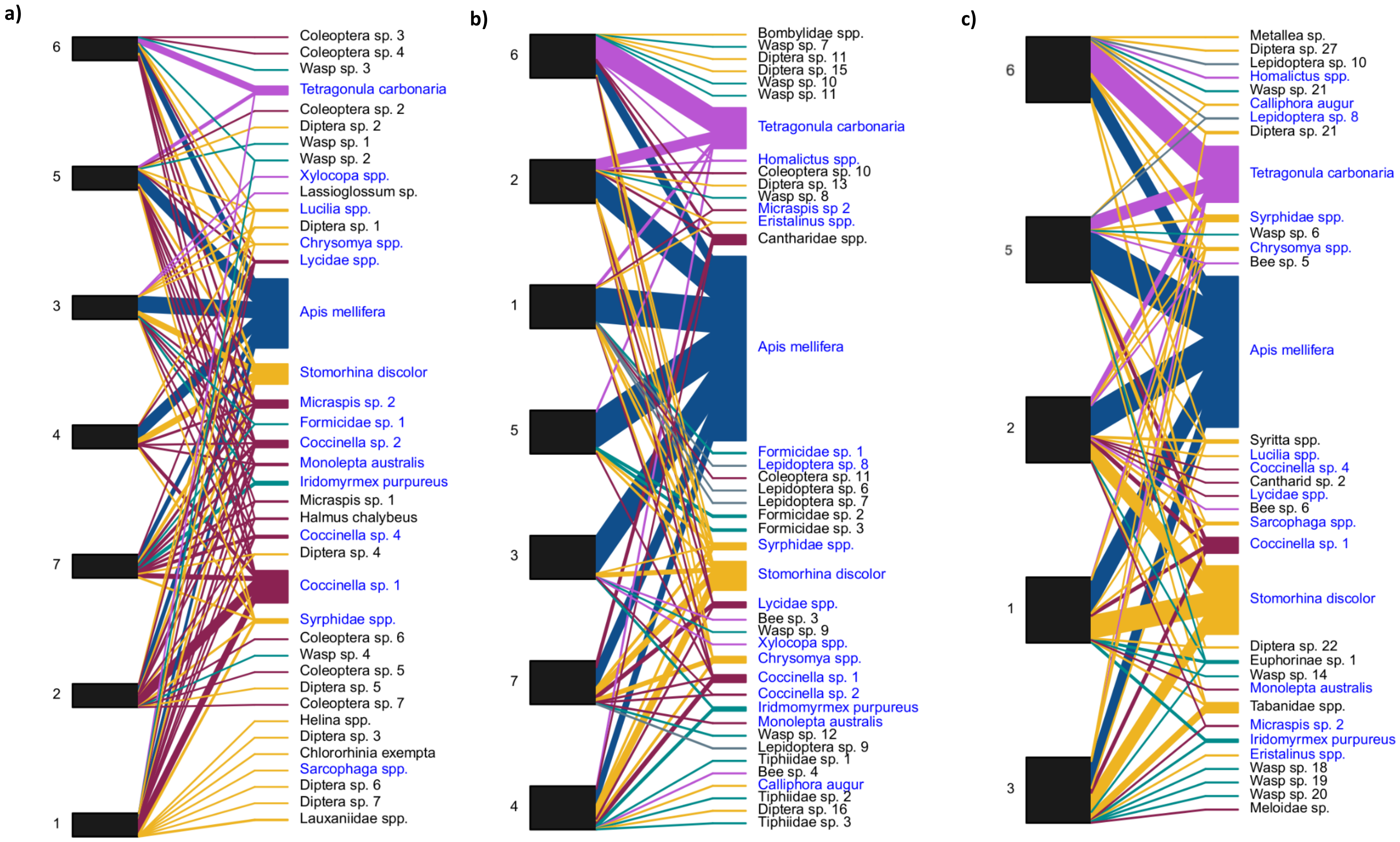

SI Figure 2: Avocado orchard site visitation networks across three years a) 2015; b) 2016; and c) 2017 (sites 4 and 7 were not surveyed in 2017). Black nodes represent orchard sites and blue text indicates species groups present across all three years.

a)

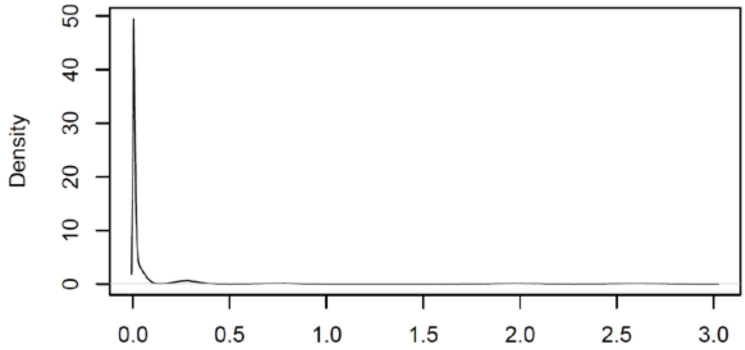

Apis mellifera (observed species strength = 2.93, *p < 0.001)*

Coccinella morphosp. 1 (observed species strength = 1.36, p-value = 0.015)

b)

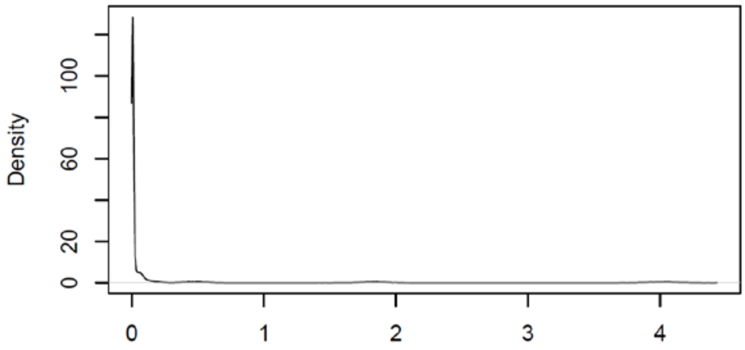

Apis mellifera (observed species strength = 4.24, $p < 0.001$)

c)

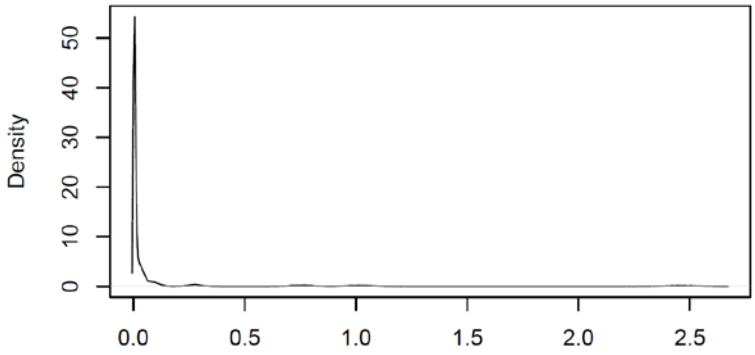

Apis mellifera (observed species strength = 2.3, $p < 0.001$)

Stomorhina discolor (observed species strength = 1.04, $p = 0.046$)

SI Figure 3: Null model distributions for species strength values (x axis) in avocado visitation pollinator networks and significant observed species in: a) 2015; b) 2016; and c) 2017.

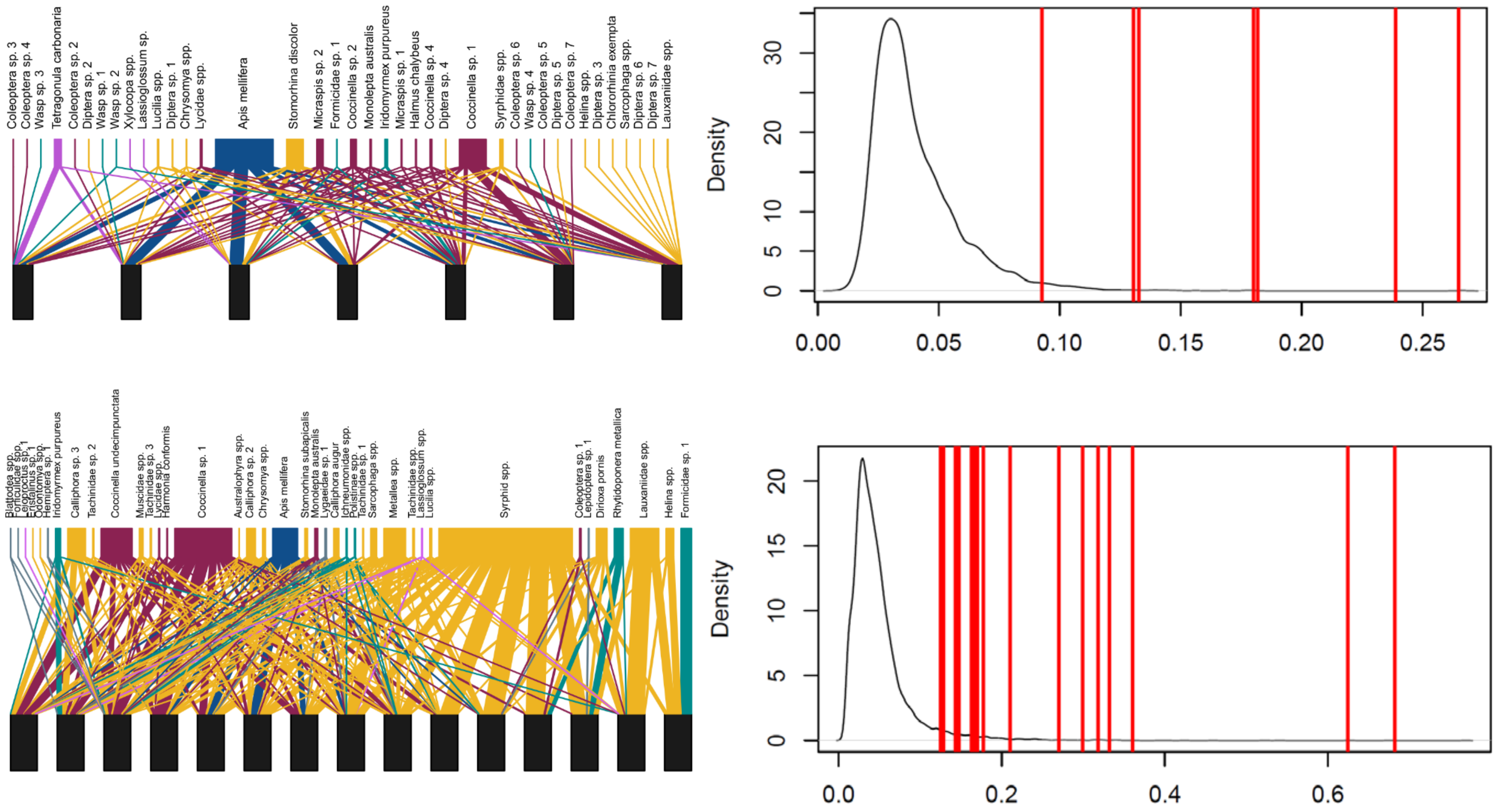

SI Figure 4: Visitation network and null distribution models for orchard site specialisation (d’). Red lines indicate observed orchard site specialisation (d’) values. Region, crop & year: Top pair) Bundaberg avocado, 2015 and Bottom pair) Sunraysia avocado, 2015.

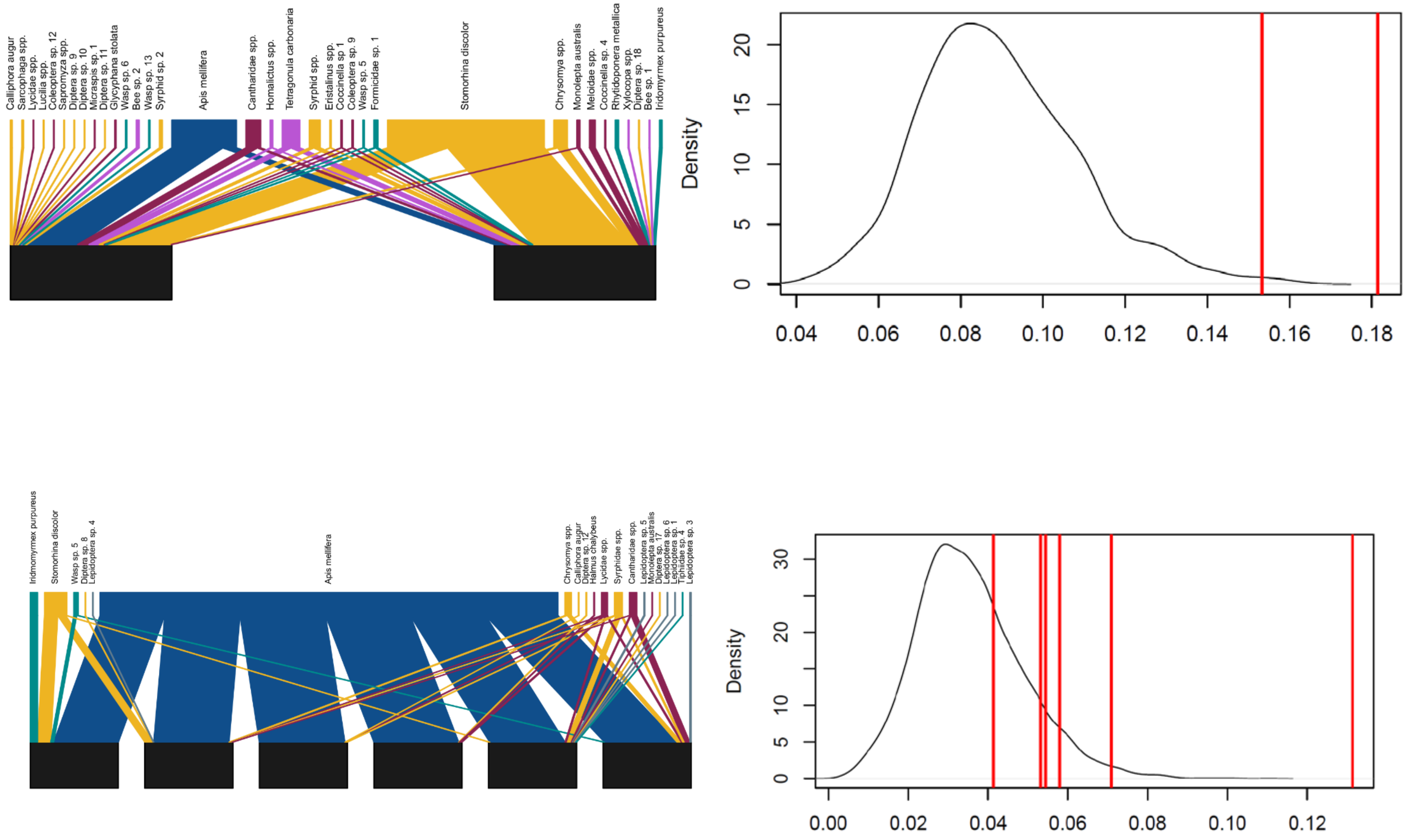

SI Figure 5: Visitation network and null distribution models for orchard site specialisation (d'). Red lines indicate observed orchard site specialisation (d') values. Region, crop & year: Top pair) Bundaberg mango, 2016 and Bottom pair) Bundaberg macadamia, 2016.

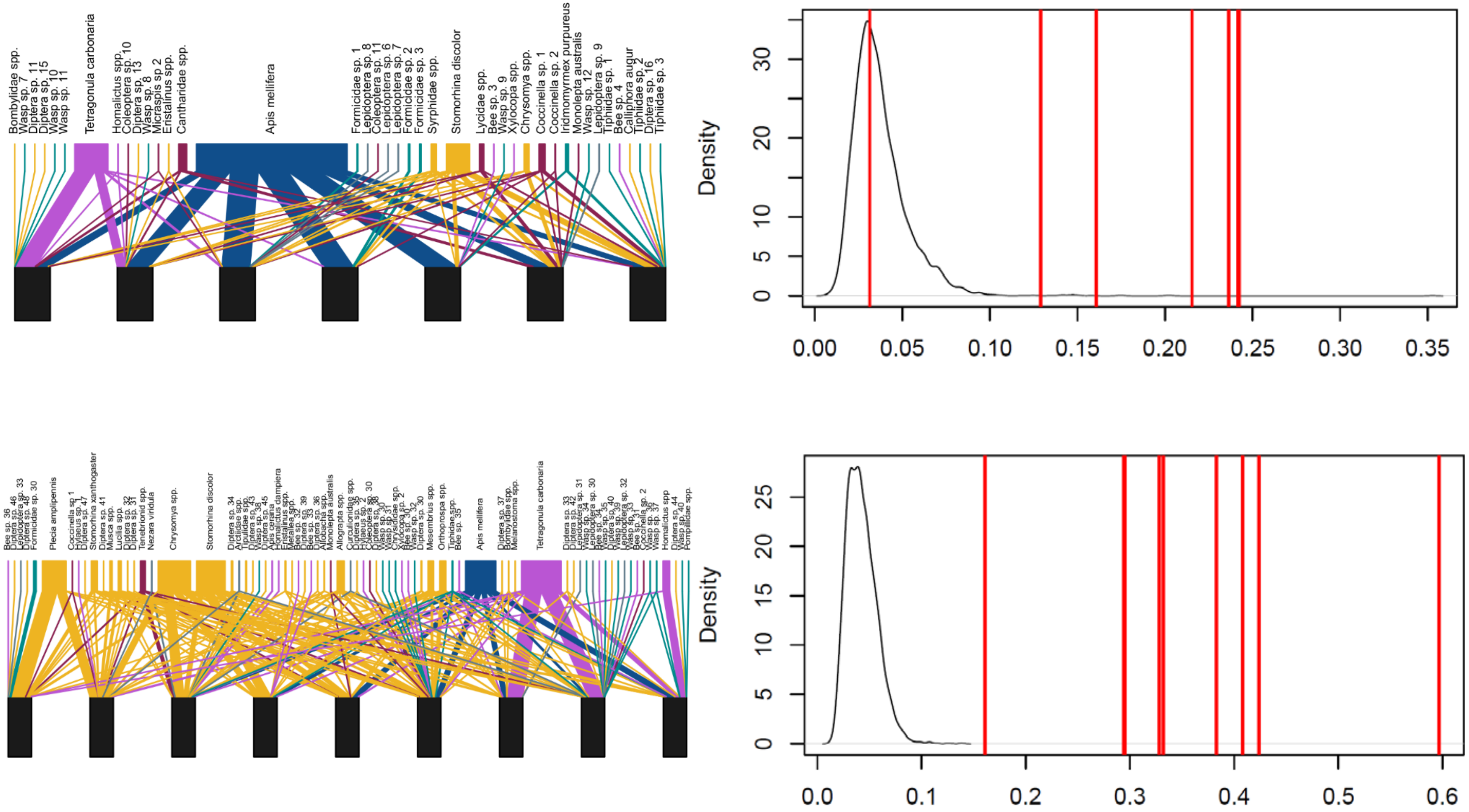

SI Figure 6: Visitation network and null distribution models for orchard site specialisation (d’). Red lines indicate observed orchard site specialisation (d’) values. Region, crop & year: Top pair) Bundaberg avocado, 2016; and Bottom pair) Mareeba mango, 2016.

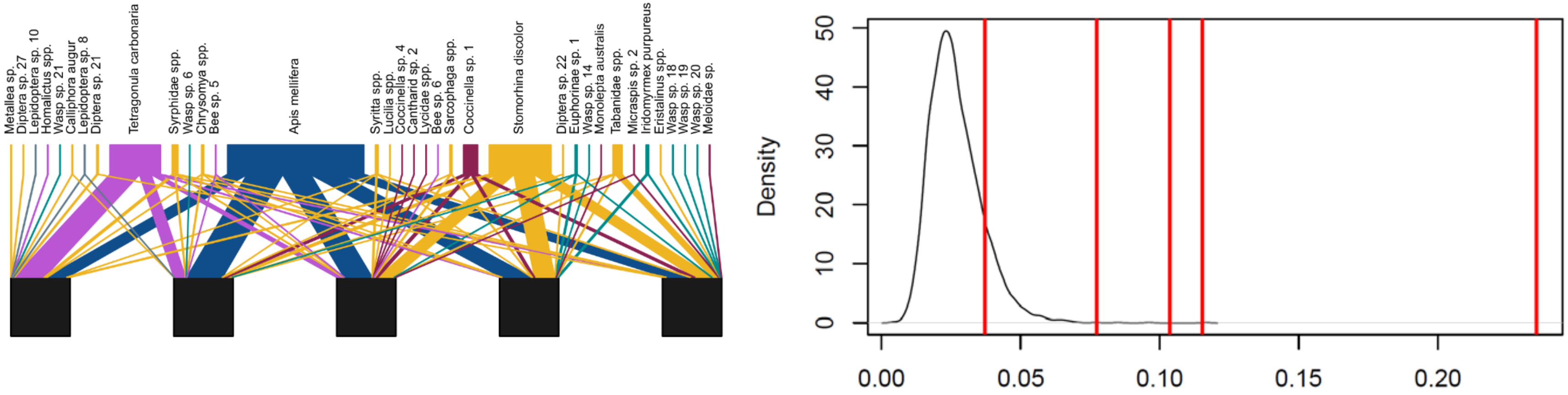

SI Figure 7: Visitation network and null distribution models for site level specialisation (d’). Red lines indicate observed site level specialisation (d’) values. Region, crop & year: Bundaberg avocado, 2017.

SI Table 3: Minimum adequate GLM models describing the influence of landscape composition on orchard site specialisation (d').

Predictor variables	Estimate	Std. Error	t value	Pr(>|t|)
Avocado – Sunraysia, 2015, 500m				
(Intercept)	-1.147934	0.903754	-1.270	0.245
Native vegetation (grazing)	-0.005833	0.027148	-0.215	0.836
Irrigated perennial horticulture	0.004830	0.012232	0.395	0.705
Avocado – Sunraysia, 2015, 1km				
(Intercept)	-1.17545	1.11182	-1.057	0.331
Conservation areas	-0.01813	0.03519	-0.515	0.625
Native vegetation (grazing)	0.00103	0.02584	0.04	0.97
Irrigated perennial hortculture	0.00702	0.01733	0.406	0.699
Avocado – Sunraysia, 2015, 2km				
(Intercept)	0.04744	2.38913	0.02	0.985
Conservation areas	0.0016	0.02262	0.071	0.946
Native vegetation (grazing)	-0.0012	0.01907	-0.063	0.952
Irrigated perennial hortculture	0.00535	0.02058	0.26	0.803
Avocado – Bundaberg, 2015, 500m				
(Intercept)	-8.39696	3.92085	-2.142	0.166
Native vegetation (grazing)	0.06869	0.04033	1.703	0.231
Irrigated perennial horticulture	0.08460	0.04743	1.784	0.216
Native forests (production)	0.08098	0.04218	1.920	0.195
Irrigated cropping	0.06392	0.03920	1.630	0.245
Avocado – Bundaberg, 2015, 1km				
(Intercept)	-4.38383	1.57379	-2.786	0.108
Native vegetation (grazing)	0.03000	0.01739	1.725	0.227
Irrigated perennial horticulture	0.04364	0.02464	1.771	0.219
Native forests (production)	0.03213	0.01681	1.912	0.196
Irrigated cropping	0.02455	0.01620	1.516	0.269
Avocado – Bundaberg, 2016, 500m				
(Intercept)	1.21419	5.84911	0.208	0.855
Native vegetation (grazing)	-0.03652	0.06197	-0.589	0.615
Irrigated perennial horticulture	-0.03036	0.07326	-0.414	0.719
Native forests (production)	-0.01407	0.06223	-0.226	0.842
Irrigated cropping	-0.02715	0.05791	-0.469	0.685
Avocado – Bundaberg, 2016, 1km				
(Intercept)	-1.292805	2.967391	-0.436	0.706
Native vegetation (grazing)	-0.008103	0.033530	-0.242	0.832
Irrigated perennial horticulture	0.008203	0.051521	0.159	0.888
Native forests (production)	0.002983	0.031919	0.093	0.934
Irrigated cropping	-0.006922	0.031383	-0.221	0.846
Avocado – Bundaberg, 2016, 2km				
(Intercept)	-2.354797	3.356879	-0.701	0.556
Native vegetation (grazing)	0.010764	0.034719	0.310	0.786
Irrigated perennial horticulture	0.014315	0.078489	0.182	0.872
Native forests (production)	0.011657	0.037924	0.307	0.788
Irrigated cropping	0.004451	0.040646	0.110	0.923

SI Table 3: Continued.

Predictor variables	Estimate	Std. Error	t value	Pr(>|t|)
Avocado – Bundaberg, 2017, 500m				
(Intercept)	-2.715371	3.450610	-0.787	0.576
Native vegetation (grazing)	0.001647	0.042694	0.039	0.975
Irrigated perennial horticulture	0.005365	0.045634	0.118	0.925
Irrigated cropping	0.016407	0.034788	0.472	0.719
Avocado – Bundaberg, 2017, 1km				
(Intercept)	-2.710611	3.687194	-0.735	0.596
Native vegetation (grazing)	0.006771	0.044618	0.152	0.904
Irrigated perennial horticulture	0.003371	0.069696	0.048	0.969
Irrigated cropping	0.010210	0.035670	0.286	0.823
Avocado – Bundaberg, 2017, 2km				
(Intercept)	-3.53326	3.04285	-1.161	0.453
Native vegetation (grazing)	0.01304	0.03115	0.419	0.748
Irrigated perennial horticulture	0.03101	0.09590	0.323	0.801
Irrigated cropping	0.01961	0.03269	0.600	0.656
Macadamia – Bundaberg, 2016, 500m				
(Intercept)	-1.222288	1.412330	-0.865	0.478
Native vegetation (grazing)	0.003163	0.016045	0.197	0.862
Irrigated perennial horticulture	-0.017177	0.015608	-1.101	0.386
Irrigated cropping	-0.020907	0.014461	-1.446	0.285
Macadamia – Bundaberg, 2016, 2km				
(Intercept)	0.432591	6.638195	0.065	0.954
Native vegetation (grazing)	0.003365	0.072490	0.046	0.967
Irrigated perennial horticulture	-0.032559	0.074075	-0.440	0.703
Irrigated cropping	-0.044929	0.079847	-0.563	0.630
Mango – Mareeba, 2016, 500m				
(Intercept)	0.260178	0.811863	0.320	0.762
Native vegetation (grazing)	-0.002735	0.009564	-0.286	0.786
Irrigated perennial horticulture	-0.023709	0.013786	-1.720	0.146
Irrigated cropping	0.020715	0.016421	1.261	0.263
Mango – Mareeba, 2016, 2km				
(Intercept)	-1.432893	1.990086	-0.720	0.504
Native vegetation (grazing)	0.001270	0.026940	0.047	0.964
Irrigated perennial horticulture	0.008398	0.025074	0.335	0.751
Irrigated cropping	0.040213	0.055415	0.726	0.501

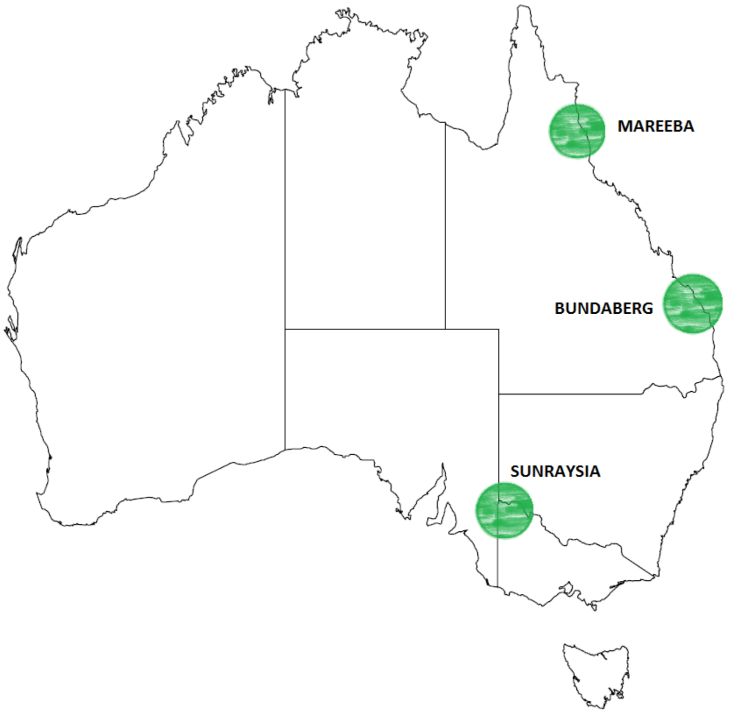

SI Figure 8: Map indicating study regions.

SI Table 4. Pollinator-crop visitation survey details.

Crop	Region	Year	Cultivar	Sampling Period	No. Orchard Sites	No. Surveys	Temp °C min-max	Humidity % min-max
Avocado	Bundaberg	2015	Hass	12 – 29 Sept	7	45	20.6 – 28.6	24.0 – 70.5
	Bundaberg	2016	Hass	12 – 27 Sept	7	47	20.3 – 29.1	25.2 – 84.2
	Bundaberg	2017	Hass	30 Aug – 15 Sept	5	42	16.2 – 33.3	13.3 – 70.0
	Sunraysia	2015	Hass	9 – 28 Oct	16	50	16.4 – 35.4	17.0 – 66.0
Macadamia	Bundaberg	2016	741	26 – 30 Sept	6	39	21.4 – 29.4	20.5 – 65.4
Mango	Bundaberg	2016	Calypso	11 – 19 Sept	2	15	21.4 – 28.8	29.4 – 67.6
	Mareeba	2016	Keitt	3 – 31 Aug	9	100	12.7 – 33.1	26.5 – 82.0

SI Table 5: Pollinator groupings.

Field visitor groups	Taxonomic family	Genera/species of identified voucher specimens	Region
Syrphid spp.	Syrphidae	*Simosyrphus, Neoplesia, Cyphipeltus*	Bundaberg
Syrphid spp.	Syrphidae	*Simosyrphus, Neoplesia*	Sunraysia
Tachinidid spp.	Tachindae	3 species	Sunraysia
Homalictus spp.	Halictidae	*Urbanus, Flindersi, Murrayi*	Mareeba

SI Table 6: Mean SVD values for different visitors. Mean ± se (n samples).

	Crop		
	**Avocado**	**Mango**	**Macadamia**
Control stigmas	0.18 ± 0.4 (410)	1.8 ± 0.3 (475)	0.16 ± 0.1 (13)
Apis mellifera	1.6 ± 0.45 (86)	0.85 ± 0.55 (41)	0.52 ± 0.21 (31)
Tetragonula carbonaria	2.67 ± 0.67 (34)	5.18 ± 3.18 (58)	NA
Stomorhina discolor	1.2 ± 0.38 (39)	2.42 ± 1.19 (50)	0.42 ± 0.19 (12)
Others	0.12 ± 0.14 (23)	0.38 ± 0.91 (17)	0.42 ± 0.19 (12)

AUSTRALIAN LAND USE AND MANAGEMENT CLASSIFICATION Version 8 (October 2016)

1 Conservation and Natural Environments

1.1.0 Nature conservation
- 1.1.1 Strict nature reserves
- 1.1.2 Wilderness area
- 1.1.3 National park
- 1.1.4 Natural feature protection
- 1.1.5 Habitat/species management area
- 1.1.6 Protected landscape
- 1.1.7 Other conserved area

1.2.0 Managed resource protection
- 1.2.1 Biodiversity
- 1.2.2 Surface water supply
- 1.2.3 Groundwater
- 1.2.4 Landscape
- 1.2.5 Traditional indigenous uses

1.3.0 Other minimal use
- 1.3.1 Defence land - natural areas
- 1.3.2 Stock route
- 1.3.3 Residual native cover
- 1.3.4 Rehabilitation

Minimum level of land use attribution in Queensland

2 Production from Relatively Natural Environments

2.1.0 Grazing native vegetation

2.2.0 Production native forests
- 2.2.1 Wood production forestry
- 2.2.2 Other forest production

3 Production from Dryland Agriculture and Plantations

3.1.0 Plantation forests
- 3.1.1 Hardwood plantation forestry
- 3.1.2 Softwood plantation forestry
- 3.1.3 Other forest plantation
- 3.1.4 Environmental forest plantation

3.2.0 Grazing modified pastures
- 3.2.1 Native/exotic pasture mosaic
- 3.2.2 Woody fodder plants
- 3.2.3 Pasture legumes
- 3.2.4 Pasture legume/grass mixtures
- 3.2.5 Sown grasses

3.3.0 Cropping
- 3.3.1 Cereals
- 3.3.2 Beverage and spice crops
- 3.3.3 Hay and silage
- 3.3.4 Oilseeds
- 3.3.5 Sugar
- 3.3.6 Cotton
- 3.3.7 Alkaloid poppies
- 3.3.8 Pulses

3.4.0 Perennial horticulture
- 3.4.1 Tree fruits
- 3.4.2 Olives
- 3.4.3 Tree nuts
- 3.4.4 Vine fruits
- 3.4.5 Shrub berries and fruits
- 3.4.6 Perennial flowers and bulbs
- 3.4.7 Perennial vegetables and herbs
- 3.4.8 Citrus
- 3.4.9 Grapes

3.5.0 Seasonal horticulture
- 3.5.1 Seasonal fruits
- 3.5.2 Seasonal flowers and bulbs
- 3.5.3 Seasonal vegetables and herbs

3.6.0 Land in transition
- 3.6.1 Degraded land
- 3.6.2 Abandoned land
- 3.6.3 Land under rehabilitation
- 3.6.4 No defined use
- 3.6.5 Abandoned perennial horticulture

4 Production from Irrigated Agriculture and Plantations

4.1.0 Irrigated plantation forests
- 4.1.1 Irrigated hardwood plantation forestry
- 4.1.2 Irrigated softwood plantation forestry
- 4.1.3 Irrigated other forest plantation
- 4.1.4 Irrigated environmental forest plantation

4.2.0 Grazing irrigated modified pastures
- 4.2.1 Irrigated woody fodder plants
- 4.2.2 Irrigated pasture legumes
- 4.2.3 Irrigated legume/grass mixtures
- 4.2.4 Irrigated sown grasses

4.3.0 Irrigated cropping
- 4.3.1 Irrigated cereals
- 4.3.2 Irrigated beverage and spice crops
- 4.3.3 Irrigated hay and silage
- 4.3.4 Irrigated oilseeds
- 4.3.5 Irrigated sugar
- 4.3.6 Irrigated cotton
- 4.3.7 Irrigated alkaloid poppies
- 4.3.8 Irrigated pulses
- 4.3.9 Irrigated rice

4.4.0 Irrigated perennial horticulture
- 4.4.1 Irrigated tree fruits
- 4.4.2 Irrigated olives
- 4.4.3 Irrigated tree nuts
- 4.4.4 Irrigated vine fruits
- 4.4.5 Irrigated shrub berries and fruits
- 4.4.6 Irrigated perennial flowers and bulbs
- 4.4.7 Irrigated perennial vegetables and herbs
- 4.4.8 Irrigated citrus
- 4.4.9 Irrigated grapes

4.5.0 Irrigated seasonal horticulture
- 4.5.1 Irrigated seasonal fruits
- 4.5.2 Irrigated seasonal flowers and bulbs
- 4.5.3 Irrigated seasonal vegetables and herbs
- 4.5.4 Irrigated turf farming

4.6.0 Irrigated land in transition
- 4.6.1 Degraded irrigated land
- 4.6.2 Abandoned irrigated land
- 4.6.3 Irrigated land under rehabilitation
- 4.6.4 No defined use - irrigation
- 4.6.5 Abandoned irrigated perennial horticulture

5 Intensive Uses

5.1.0 Intensive horticulture
- 5.1.1 Production nurseries
- 5.1.2 Shadehouses
- 5.1.3 Glasshouses
- 5.1.4 Glasshouses - hydroponic
- 5.1.5 Abandoned intensive horticulture

5.2.0 Intensive animal production
- 5.2.1 Dairy sheds and yards
- 5.2.2 Feedlots
- 5.2.3 Poultry farms
- 5.2.4 Piggeries
- 5.2.5 Aquaculture
- 5.2.6 Horse studs
- 5.2.7 Saleyards/stockyards
- 5.2.8 Abandoned intensive animal production

5.3.0 Manufacturing and industrial
- 5.3.1 General purpose factory
- 5.3.2 Food processing factory
- 5.3.3 Major industrial complex
- 5.3.4 Bulk grain storage
- 5.3.5 Abattoirs
- 5.3.6 Oil refinery
- 5.3.7 Sawmill
- 5.3.8 Abandoned manufacturing and industrial

5.4.0 Residential and farm infrastructure
- 5.4.1 Urban residential
- 5.4.2 Rural residential with agriculture
- 5.4.3 Rural residential without agriculture
- 5.4.4 Remote communities
- 5.4.5 Farm buildings/infrastructure

5.5.0 Services
- 5.5.1 Commercial services
- 5.5.2 Public services
- 5.5.3 Recreation and culture
- 5.5.4 Defence facilities - urban
- 5.5.5 Research facilities

5.6.0 Utilities
- 5.6.1 Fuel powered electricity generation
- 5.6.2 Hydro electricity generation
- 5.6.3 Wind electricity generation
- 5.6.4 Solar electricity generation
- 5.6.5 Electricity substations and transmission
- 5.6.6 Gas treatment, storage and transmission
- 5.6.7 Water extraction and transmission

5.7.0 Transport and communication
- 5.7.1 Airports/aerodromes
- 5.7.2 Roads
- 5.7.3 Railways
- 5.7.4 Ports and water transport
- 5.7.5 Navigation and communication

5.8.0 Mining
- 5.8.1 Mines
- 5.8.2 Quarries
- 5.8.3 Tailings
- 5.8.4 Extractive industry not in use

5.9.0 Waste treatment and disposal
- 5.9.1 Effluent pond
- 5.9.2 Landfill
- 5.9.3 Solid garbage
- 5.9.4 Incinerators
- 5.9.5 Sewage/sewerage

6 Water

6.1.0 Lake
- 6.1.1 Lake - conservation
- 6.1.2 Lake - production
- 6.1.3 Lake - intensive use
- 6.1.4 Lake - saline

6.2.0 Reservoir/dam
- 6.2.1 Reservoir
- 6.2.2 Water storage - intensive use/farm dams
- 6.2.3 Evaporation basin

6.3.0 River
- 6.3.1 River - conservation
- 6.3.2 River - production
- 6.3.3 River - intensive use

6.4.0 Channel/aqueduct
- 6.4.1 Supply channel/aqueduct
- 6.4.2 Drainage channel/aqueduct
- 6.4.3 Stormwater

6.5.0 Marsh/wetland
- 6.5.1 Marsh/wetland - conservation
- 6.5.2 Marsh/wetland - production
- 6.5.3 Marsh/wetland - intensive use
- 6.5.4 Marsh/wetland - saline

6.6.0 Estuary/coastal waters
- 6.6.1 Estuary/coastal waters - conservation
- 6.6.2 Estuary/coastal waters - production
- 6.6.3 Estuary/coastal waters - intensive use

SI Figure 9: ALUM Classification Scheme (ABARES 2016, The Australian Land Use and Management Classification Version 8, Australian Bureau of Agricultural and Resource Economics and Sciences, Canberra. CC BY 3.0.).
